# Dental vulnerability scale in primary health care: evidence of content and structure internal validity

**DOI:** 10.1186/s12903-021-01742-6

**Published:** 2021-08-28

**Authors:** Danielle da Costa da Palacio, Flavio Rebustini, Daniele Boina de Oliveira, João Peres Neto, Wander Barbieri, Thais Paragis Sanchez, Ana Carolina Cintra Nunes Mafra, Daiana Bonfim, Camila Nascimento Monteiro, Valmir Vanderlei Gomes Filho, Danielle Viana Ribeiro, Leandro Marsico Loschiavo, João Luiz Miraglia, Antonio Carlos Pereira

**Affiliations:** 1 CEPPAR, Hospital Israelita Albert Einstein, Av. Albert Einstein 627, São Paulo, SP Brazil; 2 Faculdade de Odontologia de Piracicaba, Universidade Estadual de Campinas, Av. Limeira, 901 - Areião, Piracicaba, SP Brazil; 3 Ciências e Humanidades – Rua Arlindo Béttio, Universidade de São Paulo - Escola de Artes, 1000 - Ermelino Matarazzo, São Paulo, SP Brazil

**Keywords:** Oral health, Dentistry, Validity study, Psychometry, Health vulnerability, Primary health care, Questionnaire

## Abstract

**Background:**

Access to oral health services remains a challenge in the Brazilian healthcare system, especially in the primary health care setting, where the use of a risk stratification tool that could identify individuals with higher dental vulnerability would be extremely valuable. However, there literature on this theme is scarce, and there is no validated instrument in Brazil that is capable of measuring dental vulnerability. Hence, this psychometric study aimed at the development and evaluation of content and internal structure validity of the Dental Vulnerability Scale for Primary Health Care (PHC).

**Methods:**

The items were developed based on a qualitative exploratory analysis. A total of 172 items were prepared and submitted to a panel of specialists, with content validity analyzed with the Content Validity Ratio (CVR), resulting in an the initial version of the instrument composed by 41 items. Internal structure validity was analyzed by Exploratory Factor Analysis (EFA), Confirmatory Factor Analysis (CFA), and by applying 3 reliability indicators (Cronbach’s Alpha, McDonald's Omega and Greatest Lower Bound – GBL), with a sample of 1227 individuals.

**Results:**

The final configuration indicated a scale of 15 items divided into 4 dimensions (overall health, oral health, infrastructure, and healthcare services) with explained variance of 72.11%. The factor loads varied from 0.37 to 0.96. The model adjustment indices were set at × 2/df_(51)_ = 3.23, NNFI = 0.95, CFI = 0.98, GFI = 0.96, AGFI = 0.97, RMSEA = 0.04 and RMSR = 0.03.

**Conclusion:**

DVS presented satisfactory evidence of validity, indicating its suitability to be used by healthcare professionals, students and managers to plan oral health actions and services at PHC.

## Background

Oral health has been growing and strengthening itself as an integral and inseparable part of public health policies, based on the guidelines proposed by the Brazilian Ministry of Health, by means of a model of care that seeks to expand access to services through the Oral Health Teams (OHT) included in the Family Health Strategy (FHS) [1, 2]. Brazil is a large country with great territorial and populational extension (approximately 210 million inhabitants), and it is the only country in the world with more than 200 million inhabitants to include the right to health as constitutional and universal. However, it is known that equalizing access and guaranteeing the integrality of actions is only possible through the organization of care based on population management [3, 4].

Despite the expressive increase in the implementation of OHT within the FHS over the years, i.e., superior than 470% within a 14-year period, and the growth in population coverage, 57% of the population still has no access to dental care. Therefore, an instrument that enables identification of the primary sources of vulnerability amidst the reality of millions of people can change this scenario, strengthen work management, and propose an equanimous offer in the access to oral health [3].

Nevertheless, equity in access remains a challenge to be resolved, in the context of considerable limited resources and a repressed demand, with services seeking strategies to prioritize individuals with the greatest needs in oral health [5, 6]. For the identification of oral health needs, it is essential to discuss the concepts of risk and vulnerability, two distinct concepts that are commonly addressed together [7–9].

The definition of risk is related to the probability of hazard, while vulnerability is defined as a characteristic of who or what is vulnerable or is fragile [10]. Vulnerability develops from the grouping of multiple risk factors that mutually reinforce each other, instigated by the lack of material, social, and environmental resources, which are indispensible for the individual’s well-being [11]. Some factors, such as age, ethnicity, working status, family income, schooling level, conditions of access to healthcare services, living conditions, and others, determine the individual’s greater or lesser susceptibility to harm and, consequently, higher or lower vulnerability [12]. The lowest vulnerability is represented by resilience and resistance of the individuals and communities when exposed to dangerous processes or events, by the so-called protection factors, such as a high educational level, economic stability, and increase in physical activity, among others [10, 12].

An association between vulnerability and oral health morbidity has been found in the poorest and most vulnerable regions of Brazil. Thus, the ability to measure vulnerability is recognized as an important element for the effective reduction of risk, and the use of instruments such as assessment scales could lead to a more equitable access of priority services [7, 10, 11], helping with the organization of the healthcare system.

Therefore, the use and impact of these types of questionnaires and scales have been evaluated before by Coelho and Savassi Scale [13–16], with the aim of prioritizing home visits, or by other authors [5], as a programming tool for the family's oral health teams, based on the social information (type of housing and maternal education) found in the Primary Care Information System.

This type of assessment instrument should measure or evaluate an object, so it is important that they have accumulated robust evidence of validity, and they should present a simple, clear language and cultural equivalence [17]. No scientifically validated instrument capable of measuring dental vulnerability was found in the literature [18]. Hence, this work aimed at developing and searching for evidence of content validity and internal structure of the Dental Vulnerability Scale (EVO) for PHC considering the Brazilian reality.

## Methods

This is a psychometric study focused on the evaluation of evidence of content validity and internal structure, following current recommendation of the American Educational Research Association (AERA), American Psychological Association (APA), and the National Council on Measurement in Education (NCME) [19]. This study was approved by the Research Ethics Committee of the Municipal Health Department of São Paulo (Protocol 97371218.4.0000.0086).

The study was conducted in two stages, the first to evaluate content validity, and the second the internal structure of the DVS. Since there is no clear definition for vulnerability, initially a qualitative exploratory analysis was made to detect factors that could be associated with vulnerability, which in turn subsidized the development of the items of the instrument. According to DeVellis [20], a loss of 50% of items is expected during the validation process, therefore it was opted for an extensive stage to formulate the items.

### Stage 1: Evidence of content validity

This stage utilized a convenience sample composed of 188 specialists (dental surgeons, oral health technicians, oral health assistants, undergraduate dental students, physicians, nurses, licensed practical nurses, and social workers) from all regions of Brazil, of both sexes, aged over 18 years, who digitally signed the Informed Consent Form, and filled out the research questionnaire received, which had as key question, “if you have to measure dental vulnerability, which risk factors would you consider?”. At this stage, content analysis was applied with the purpose of synthesizing the various meanings reported by the respondents. Bardin’s [21] methodological orientations were used.

The questionnaire was sent to specialists through a RedCap® link, reported via WhatsApp® application and by email, to groups that work directly or indirectly with oral health. The link sent could be forwarded to other specialists, the snowball method [22] was used to reach a larger number of participants in this stage of the study, with original recipients of the RedCap® being able to forward it.

To ensure reliability of the classification performed through the content analysis, four external and impartial judges were invited to review credibility of the categories established by the researchers. The items developed from the previous stage were submitted to a panel of 40 judges, who were asked to evaluate the relevance (item that checks dental vulnerability) and the clarity of the items (if the item was correctly written), besides allowing possible suggestions for improving the wording of the items. The Content Validity Ratio (CVR) developed by Lawshe [23] was applied to the analysis of the responses of the judges. Adoption of the CVR aimed at a more rigorous application of the content validity index, and allowed the adoption of a larger number of judges. The recommendation by Lynn [24] indicates that a judge panel size of five would be adequate, however more recent recommendations by Epstein et al. [25] specifies that the panel of judges should be composed of researchers, translators, professionals, methodologists, and lay persons, while Gilbert and Prion [26] emphasizesg that such panels should have specialists from different professional levels. Taking in to account these more recent recommendations, and the fact that Brazil is a country of continental dimensions, with a wide cultural diversity, it would have been risky to restrict opinions to only five experts, which could have biased the analysed.

### Stage 2: Evidence of internal structure validity

The scale was answered by dental offices, in other Primary Care Units facilities, and in other home visits by field researchers trained at the administrative districts of Vila Andrade and Campo Limpo, located in the South Zone of São Paulo, with a estimated population of 387,408 individuals with 75% covered by the FHS. According to a survey conducted by the SEADE Foundation in 2019 [27], and data from the SIAB [28], 54.4% this population do not have private health insurance, and 58.9% depends exclusively on the Unified Healthcare System (SUS) [Brazilian National Public Health System].

To characterize the population studied, the “Brazil Classification Criterion” [29] and the individual and family registration forms of the E-SUS [30], were applied, in addition to the ICF.

### Sample

The sample size of psychometric studies is usually estimated based on the number of items [31, 32] that demonstrate a ratio of 20:1 or more; that is, 20 respondents for each item of the instrument would be the ideal [33]. In this sample, a ratio of 29.9 persons/item was established, which preliminarily ensured that the sample size would be not responsible for possible inaccuracies in the analyses.

### Statistical analysis

#### Exploratory factor analysis (EFA) and confirmatory factor analysis (CFA)

Performance of the exploratory and confirmatory factor analyses requires the fulfillment of various stages, such as data inspection, factor analysis, factor retention technique, factor rotation technique, and factor load cut-off level [34]. These stages are systematically presented for validation of internal structure.

The dimensionality of the instrument was tested by applying the Robust Parallel Analysis (RPA) through Optimal Implementation of Parallel Analysis (PA) [35] with the Minimum Rank Factor Analysis (MRFA) that minimizes the common variation of the residues [36]. The robustness of the test was determined from the association of a bootstrap with an extrapolation of the sample to 5000.

The Parallel Analysis is considered one of the most robust and precise techniques used to test dimensionality [37–39]. The factors were extracted with robust Unweighted Least Squares (ULS) that reduce residues of matrices [31]. Non-orthogonal Promax rotation was applied [40].

The adequacy of the models for the exploratory and confirmatory factor analysis was evaluated taking into consideration the number of participants and the levels of the parameters that indicate an excellent explanation of the model. Thus, the minimum factor loads must be above 0.30; communalities with values above 0.40, and explained variance close to or greater than 60% [33, 41]. The maintenance or removal of items was done considering the explained variance of the model; factor load values and communalities below the minimum established.

For the CFA, the maximal likelihood method of extraction was adopted as a reference for model quality, using the cut-off techniques established by Sivo et al. [42]. The indices Goodness-of-Fit (GOFs): robust Mean and Variance-adjusted Chi-square/df (X2/df < 5) [43]; Non-Normed Fit Index (NNFI > 0.93); Comparative Fit Index (CFI > 0.94); Goodness-of-Fit Index (GFI > 0.95); Adjusted Goodness-of-Fit Index (AGFI > 0.93); Root Mean Square Error of Approximation (RMSEA < 0.07), and Root Mean Square of Residuals (RMSR < 0.08) were adopted [42].

### Reliability

Three indicators were used: Cronbach’s Alpha [44], McDonald's Omega [45] and the Greatest Lower Bound—GBL [46]. The implementation of multiple reliability indicators aimed at reinforcing the model adjustment. In addition, various previous published articles have shown the inconsistency of reliability by means of Cronbach’s Alpha [47–50]. Alpha values greater than 0.90 should not be sought, since they can indicate duplication of content of the items and point towards redundancy more than to homogeneity [51]. Therefore, the authors chose analysis of three reliability techniques to increase precision of the study. Analyses were performed with the SPSS 23 [52] and Factor 10.10.03 [53, 54] software.

## Results

The results are reported in three stages: development of the items, evidence of content and internal structure validity.

### Stage 1: Development of the items

In the development of items for the EVO instrument, a primary questionnaire was sent to 188 oral health specialists. These experts, were mostly female (78.7%), dental surgeons (86.2%), having completed specialization degree (51.6%), and having professional experience at a PCU (79.8%), followed by work in a private office (73.9%). In order for the professional experience to be considered as adequate, this item could be marked more than once. All regions of Brazil contributed with answers, but the most expressive was the Southeast region (62.8%).

Analysis and classification of the content of the specialists’ responses for the qualitative exploratory stage resulted in the appearance of 172 factors associated with dental vulnerability, divided into 6 preliminary dimensions (healthcare services, social services, as well as services for mental health, oral health, infrastructure, and overall health). The factors were transformed into items of the instrument and prepared in an interrogative manner that enabled responses only in the dichotomous model (“0—No” and “1—Yes).

### Stage 2

### Stage 2.1 Evidence of content validity

After the development of the items, content validation was performed by 40 judges who gave their opinion on each item considering clarity, precision and relevance, and decided whether these items were suitable or not to compose the EVO. The selection of the 40 judges was made using the snowball method. The judges 60% were female, most resided in the Southeast region (92.5%), having their specialization as highest level of education concluded (52.5%). This panel was more heterogeneous, because in addition to the professionals linked to oral health (80%), other professional categories also participated, represented by physicians, nurses, speech therapists, pharmacists, and psychometrists.

The CVR establishes its critical value based on the number of judges. Therefore, for 40 judges the critical value was 0.25 [55–57]. The critical value of CVR was applied to evaluate the relevance (if the item measures the latent variable) and clarity of the items (semantics). The items that were at the critical value limit, that is, that presented with values of 0.25 for relevance, were included for the internal structure stage. The analysis resulted in the continuance of 41 of the 172 items initially developed. The result was a CVR_mean_ of 0.33 for relevance of the item, and of 0.39 for the clarity of the item. Table 1 presents the values of CVR for relevance and clarity of the items that surpassed the critical value.

**Table 1:** CVR values for the preliminary version items

	Relevance	Clarity
121. Do you have a bathroom in your house?	0.55	0.60
14. Do you consume an excessive amount of sugar?	0.50	0.10
125. Do you have sewage collection in your house?	0.50	0.60
129. Do you use a public primary care unit?	0.45	0.70
19. Are you diabetic?	0.40	0.25
44. Do you smoke?	0.40	0.40
54. Do you use drugs?	0.40	0.45
62. Do you consider your family has good hygiene habits?	0.40	0.40
65. Do you consider you have good oral health?	0.40	0.35
124. Do you have running water in your house?	0.40	0.60
128. Are you familiar with the public primary care unit where you can visit?	0.40	0.60
7. Are you bedridden?	0.35	0.25
9. Are you HIV-positive?	0.35	0.30
15. Do you consume an excessive amount of carbohydrates?	0.35	− 0.10
67. Do you have oral cancer?	0.35	0.45
68. Have you ever had oral cancer?	0.35	0.50
8. Do you live only inside the house?	0.30	0.10
20. Do you have any motor difficulty?	0.30	0.10
23. Do you have any chronic disease?	0.30	− 0.10
45. Do you have cancer?	0.30	0.45
47. Have you ever undergone radiation therapy?	0.30	0.40
51. Do you consume alcohol?	0.30	0.30
63. Is there any tooth missing in your mouth?	0.30	0.45
64. Do you believe that when a tooth has a problem. it is better to extract it?	0.30	0.35
90. Do you experience depression?	0.30	0.55
120. Do you have access to water with fluoride?	0.30	0.25
127. Do you consider your house far from the dental care service you use?	0.30	0.50
131. Are you accompanied by a Family Health oral health team?	0.30	0.50
137. Do you have a dental health insurance plan?	0.30	0.40
12. Do you have any heart disease?	0.25	0.40
13. Do you have any disabling conditions?	0.25	0.05
40. Do you have Down syndrome?	0.25	0.40
69. Do you have any decayed teeth?	0.25	0.45
71. Do you consider that it is important to care for your mouth?	0.25	0.55
82. Do you believe that oral diseases can be avoided?	0.25	0.45
83. Do you consider yourself responsible for your oral health?	0.25	0.50
84. Do you consider it important to have all the teeth in your mouth?	0.25	0.40
85. Do you chew well?	0.25	0.45
123. Do you have electricity in your house?	0.25	0.65
133. Do you see the dentist without paying?	0.25	0.50
135. Have you ever gone to the dentist for a treatment?	0.25	0.50
Mean	0.33	0.39

Making an item clear is fundamental for a good understanding of the researched phenomenon, and it is necessary to structure concise writing, avoiding tautology to not lose relevance [58]. All items considered relevant (with CVR > or equal to 0.25) were included in the preliminary version, and items with less than 0.25 of clarity were changed in the text, as suggested by the judges. Some items that had suggestions from judges and had a CVR above critical value were also changed, so that they were clearer and more understandable to the target population.

Among the changes, there is “are you diabetic?” for example, considered relevant by the judges with a CVR = 0.40, presented with a clarity CVR of 0.25, requiring a reconfiguration of the writing so that it could be part of the items sent to the field. The item previously written as “are you diabetic” was changed to “do you have diabetes”, following a suggestion of the judges to modify the writing. In the same way, the item “do you have access to fluoridated water?” with a relevance CVR of 0.30 and a clarity index of 0.25, was revised and according to content analysis, had the writing changed to “do you have access to water with fluoride?”. Another example of an item considered as showing lower clarity (CVR = 0.10) and good level of relevance (CVR = 0.30) had the objective of knowing if the study participants were bedridden or not. The question initially written “Do you live limited at home?” was modified to “Do you live only inside the house?”.

Hence, the 13 items that needed rewriting for adequacy of the clarity item (CVR < 0.25) were revised and inserted into the final field version of the proposed scale.

#### Stage 2.2: Evidence of internal structure validity

The instrument was applied to 1227 participants in the South Zone of São Paulo, in the districts of Campo Limpo and Vila Andrade, at the PCU that are part of the partnership that the *Sociedade Beneficente Israelita Brazileira Albert Einstein* (SBIBAE) has with the Municipal Health Department of São Paulo (SMS/PMSP) since 2001 for implantation, implementation, and management of FHS units and teams. There are 30 OHT, 30 dental surgeons, 23 oral health technicians, and 30 oral health assistants.

The instrument was applied by the OHT professionals, duly and previously trained on the objective and methodology of the research. Additionally, a Whatsapp® group was created with all researchers involved to answer questions, ensure a higher standard, and minimize inter-examiner errors. Data were collected simultaneously at 11 PCU by 30 OHT, in the period from September 27, 2019 to November 28, 2019, during the work routine of the professionals (scheduled visits, dental emergencies, and home visits).

#### Stage 2.3: Characterization of the participants

As per Table 2, the instrument was answered by the largest number of participants possible within the determined period for collection at the primary care units. The respondents had a mean age of 33.30 years and a standard deviation of 17.30, and there was a prevalence of females (68.2%) and brown skin color (52.9%). The majority of these responded to the questionnaire during their routine dental visit (65.2%), while the other forms of capture from the respondents were distributed among urgent consultations (20%), home visits (3.6%), triages (4.9%), groups (1.8%), and others (4.5%).

**Table 2:** Profile of respondents

Profile of respondents (n = 1227)	Description
Age (years)*	33.30 (17.30)
Place of capture of respondent (n = 1151)	Absolute no. (%)
Routine visit	751 (65.2)
Urgent consultation	230 (20.0)
Home visits	41 (3.6)
Triage	56 (4.9)
Group	21 (1.8)
Others	52 (4.5)
*Sex (n* *=* *1223)*	
Male	386 (31.6)
Female	834 (68.2)
Other	3 (0.3)
*Skin color (n* = *1199)*	
White	384 (32.0)
Black	162 (13.5)
Brown	634 (52.9)
Yellow	14 (1.2)
Indian	5 (0.4)
*Level of schooling (n* = *1225)*	
Illiterate	52 (4.2)
Incomplete elementary school	403 (32.9)
Complete elementary school	109 (8.9)
Incomplete high school	174 (14.2)
Complete high school	411 (33.6)
*Complete college (or graduate) degree*	60 (4.9)
Graduate degree	16 (1.3)
*Occupation (n* = *1224)*	
Salaried employee	323 (26.4)
Salaried domestic worker	54 (4.4)
Self-employed	175 (14.3)
Employer	4 (0.3)
Unpaid worker	6 (0.5)
Student	123 (10.0)
Retired	76 (6.2)
Unemployed	289 (23.6)
Not applicable	174 (14.2)
*Social class/Family income (Brazilian Geography and Statistics Institute (IBGE) (n* = *1219)*	
A—more than 20 minimum wages	1 (0.1)
B—from 10 to 20 minimum wages	6 (0.5)
C—from 4 to 10 minimum wages	31 (2.5)
D—from 2 to 4 minimum wages	230 (18.9)
E—up to 2 minimum wages	951 (78.0)
Number de persons who live in the house (including respondent) ** (n = 1204)	3.00 [1.00, 16.00]
*Number of rooms in the house (including living room, kitchen, and bathrooms)* ** *(n* = *1221)*	
Prevalence of decays (n = 1209)	4.00 [1.00, 14.00]
DMFT-D, total score** (n = 1037)	11.00 [0.00, 32.00]
DEFT, total score** (n = 172)	3.50 [0.00, 26.00]

^*^Continuous numerical variables described by mean and standard deviation

^**^Discrete numerical variables described by medians, minimum, and maximum values

As to the level of education, we noted that most of the participants had concluded high school (33.6%) or had not completed elementary education (32.9%); a small percentage had a salaried job (26.4%), while another portion was unemployed (23.6%). This scenario mostly pointed towards a family income of less than 2 minimum wages. As to living conditions, the majority of homes were composed of 4 rooms, and a median of 3 persons per household. In reference to oral health, the sum of teeth that were decayed, missing, and filled teeth (DMFT) had a median of 11.00 for adults, and when the same index for the adults was applied to children, which measures teeth that are decayed, extracted, and filled (DEFT), had a median of 3.50.

The initial analysis indicated that the measurements of adequacy of the sample established Kaiser–Meyer–Olkin (KMO) test of 0.65 and Bartlett test of sphericity of 8622.6 (df = 820; *p* < 0.01). The results indicate the data are adequate to be analyzed by factor analysis [33]. The correlations among the items were established between − 0.24 and 0.50, with a predominance of weak correlations, which can imply possible problems with adjustment of the model in the factor analysis.

For the initial test of dimensionality by the Parallel Analysis, the model proposed in content analysis had six dimensions. Dimensionality results indicated the existence of four dimensions for the set of items analyzed. The Parallel Analysis indicated relevant eigenvalues from 3.02 to 1.30, generating an explained variance of 36.56% for the model. Besides the dimensionality testing not confirming the six-factor criterion, only 26 out of 41items presented with factor loads higher than 0.30, and it was not possible to find an adequate alignment of these loads with the content of the initial factors. Only 13 items presented communalities superior to 0.30 (Table 3).

**Table 3:** Factor loads and communalities of the initial extraction with six dimensions

Item	F1	F2	F3	F4	F5	F6	Communality
1	− 0.05	− 0.03	− 0.04	0.06	**0**.**59**	− 0.02	0.34
2	− 0.01	− 0.02	− 0.02	− 0.02	**0**.**80**	− 0.04	0.64
3	0.00	0.06	0.01	0.01	0.14	0.03	0.03
4	0.03	0.00	0.22	− 0.03	− 0.06	0.05	0.05
5	0.02	0.00	**0**.**61**	0.02	0.09	0.01	0.41
6	− 0.02	0.20	− 0.18	0.03	0.06	0.10	0.08
7	− 0.02	0.18	− 0.08	0.02	0.11	0.09	0.06
8	− 0.03	− 0.05	**0**.**36**	0.05	− 0.14	− 0.02	0.14
9	0.00	0.02	**0**.**53**	− 0.02	0.06	0.02	0.30
10	0.00	− 0.03	**0**.**66**	0.00	− 0.05	0.00	0.42
11	0.09	− 0.01	0.03	− **0**.**34**	0.02	0.00	0.11
12	− 0.03	**0**.**34**	0.02	− 0.07	− 0.03	0.02	0.13
13	0.04	− 0.08	0.08	− 0.14	0.10	0.08	0.05
14	0.04	− 0.05	0.11	− 0.06	0.11	0.05	0.04
15	0.00	0.20	− 0.04	0.04	− 0.05	− 0.03	0.05
16	− 0.01	0.29	− 0.04	− 0.06	− 0.05	0.05	0.09
17	− 0.02	− 0.15	0.02	0.21	0.02	− 0.04	0.07
18	− 0.04	0.19	0.24	0.09	− 0.04	0.03	0.09
19	− 0.06	0.23	0.05	− 0.02	0.01	0.04	0.06
20	− 0.01	− **0**.**47**	− 0.07	0.05	0.00	0.06	0.24
21	0.02	− 0.09	0.02	− **0**.**58**	0.03	0.08	0.33
22	0.01	− 0.04	0.05	− **0**.**46**	0.01	0.09	0.21
23	− 0.01	**0**.**34**	− 0.05	0.10	− 0.02	0.00	0.13
24	0.02	− 0.02	0.04	**0**.**33**	0.04	0.10	0.14
25	0.07	− 0.07	0.03	**0**.**42**	0.02	0.06	0.21
26	− 0.02	− 0.21	0.04	**0**.**31**	0.00	0.07	0.16
27	0.00	− 0.02	0.01	**0**.**54**	0.04	0.01	0.29
28	0.05	− 0.26	− 0.25	0.02	− 0.05	0.02	0.14
29	0.02	0.07	0.23	− 0.07	0.06	− 0.02	0.07
30	0.18	− 0.11	0.01	− 0.02	− 0.06	− 0.01	0.04
31	**0**.**87**	0.04	− 0.02	0.03	− 0.01	0.00	0.78
32	**0**.**97**	0.02	0.00	− 0.06	0.02	− 0.04	0.89
33	**0**.**84**	0.00	0.00	0.01	0.00	− 0.01	0.70
34	**0**.**40**	− 0.15	0.03	− 0.06	− 0.04	0.07	0.19
35	− 0.06	0.07	− 0.03	− 0.04	0.08	− 0.05	0.02
36	0.11	0.03	− 0.01	0.18	0.01	**0**.**33**	0.20
37	0.10	0.02	0.01	0.17	− 0.01	**0**.**39**	0.24
38	− 0.07	− 0.02	− 0.01	− 0.09	0.01	**0**.**74**	0.53
39	0.00	0.05	− 0.02	− 0.08	− 0.03	**0**.**74**	0.54
40	0.03	0.00	0.09	0.04	− 0.01	0.24	0.07
41	− 0.02	− 0.16	− 0.05	− 0.04	− 0.06	− 0.11	0.05

**In bold**—factor loads > 0.30

Some GOFs of the CFA did not converge: chi-square, NNFI, CFI, and RMSEA; GFI = 0.96, AGFI = 0.95 and RMSR = 0.03 were established at appropriate levels. Nonetheless, several authors [59–61] pointed out that the presentation of the GOFs is not sufficient for the adjustment of the model. It should be clear that the factor loads, both by the confirmatory factor analysis (CFA) and the exploratory factor analysis (EFA), showed no adjustment of the model.

The inter-factor correlations remained between − 0.07 and 0.26, indicating a weak association among the dimensions of the instrument. The reliability values of the initial model, which were α of 0.58 and ω of 0.76, and GLB of 0.79, also did not show good levels. These results lose their relevance for not having model adjustment.

Faced with the non-adjustment of the model, the literature recommendation was adopted to remove the items that did not present adjustment of the model. For this, the values of factor load, communality, and cross-loading were adopted as criteria for maintenance or withdrawal of the item [33], in addition to the item content. The items were removed one by one and the analyses were redone until there was an adjustment of the internal structure, considering the statistical and content standpoints.

After removal of the items that did not reach the set of established indicators, the parallel analysis indicated a new model with four dimensions, with an explained variance of 72.11%. Measures of adequacy of the sample were also improved with the removal of items with KMO of 0.70 and Bartlett test of sphericity of 5295.1 (df = 105; *p* < 0.01). Table 4 displays the adjustment of factor loads and communalities of the new model.

**Table 4:** Factor loads and communalities of the final model with four dimensions

Item	F1	F2	F3	F4	h^2^
5. Does your health keep you from doing any daily activities?	**0**.**83**	0.04	0.03	0.01	0.43
9. Do you have any movement difficulties?	**0**.**61**	− 0.02	0.02	0.01	0.38
10. Do you have any disease that requires monitoring?	**0**.**45**	0.06	− 0.01	− 0.01	0.36
24. Do you consider it important to take care of your mouth?	0.03	**0**.**34**	0.01	0.08	0.14
25. Do you believe that diseases of the mouth can be avoided?	− 0.01	**0**.**57**	− 0.01	− 0.01	0.22
26. Do you consider yourself responsible for your oral health?	0.01	**0**.**38**	− 0.06	0.03	0.13
27. Do you consider it important to have all the teeth in your mouth?	0.02	**0**.**61**	− 0.03	− 0.06	0.30
31. Do you have a bathroom in your house?	0.02	− 0.01	**0**.**88**	0.01	0.78
32. Do you have electric power in your house?	0.01	− 0.04	**0**.**97**	− 0.03	0.89
33. Do you have running water in your house?	0.01	0.01	**0**.**86**	− 0.01	0.71
34. Do you have sewage system in your house?	0.01	− 0.01	**0**.**36**	0.08	0.15
36. Are you familiar with the public primary care unit where you can visit?	0.02	0.18	0.09	**0**.**32**	0.20
37. Do you use the public primary care unit?	− 0.01	0.21	0.08	**0**.**37**	0.24
38. Are you accompanied by an oral health team?	0.01	− 0.09	− 0.07	**0**.**74**	0.53
39. Do you see the dentist without paying?	0.01	− 0.10	0.01	**0**.**75**	0.53

F1—“Overall Health” Dimension; F2—“Oral Health” Dimension–F3; – “Infrastructure” Dimension”; and F4—“Healthcare Service” Dimension; h^2^—commonalities**; In bold—**factor loads ≥ 0.30

Saturation of factor loads in Dimension 1 varied from 0.45 to 0.83, respectively, for items 5, 9, and 10. The contents of the saturated items in this dimension are coherent, which enabled the denomination of “overall health dimension”. In items 24, 25, 26, and 27, the saturation varied between 0.34 and 0.61, denominated from the “oral health” content. The “infrastructure” dimension was defined for the grouping of items 31, 32, 33, and 34 with saturation levels between 0.36 and 0.97; while items 36 to 39 belonging to the “healthcare service” factor had a saturation of 0.32 to 0.74. The adjusted model presented no cross-loading problems and Heywood cases. Therefore, all the items of the instrument substantially saturated in a single dimension and there was no violation of the limits (− 1 to + 1) in factor loads. The confidentiality values of the initial model were α of 0.64, ω of 0.99, and GLB of 0.82. Some items in the final version had communality values below the cut-off level, but they remained because the model proved adequate for all the other indicators.

**Fig. 1:**
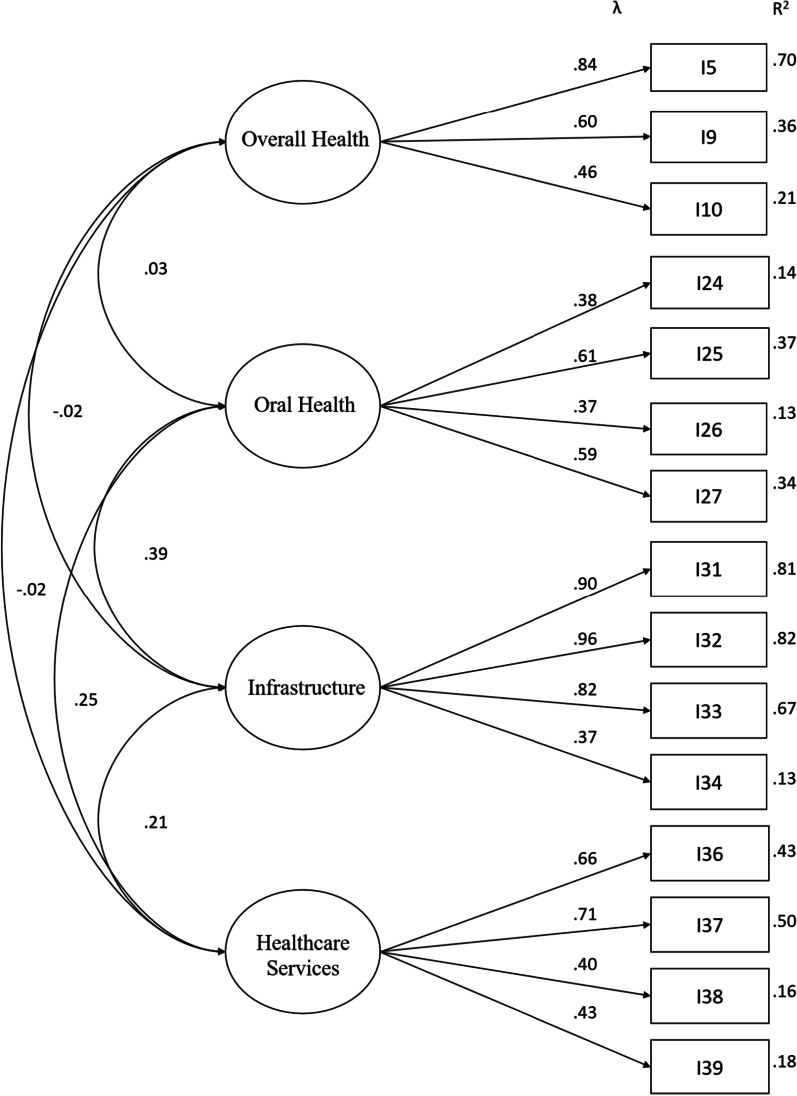
Pathy diagram of the final model of the dental scale

The GOFs of the confirmatory factor analysis indicated values of × 2/df(51) of 3.23; in that, NNFI of 0.91; CFI of 0.94; GFI of 0.96; AGFI of 0.94; RMSEA of 0.04, and RMSR of 0.03. Besides presenting the GOFs that met the established criteria, the final model presented satisfactory values of the factor loads and predictive power, shown in Fig. 1.

### Final vulnerability scale

The final model presented factor loads for the confirmatory analysis that varied from 0.37 to 0.96, with R^2^ values that ranged from 0.14 to 0.91. Therefore, both the primary indicators (factor loads and predictive value of the item) and the GOFs were established at satisfactory values. In this way, the indicators, both of EFA and CFA, assure a good solution for the 15-item model with four dimensions presented in Table 5.

**Table 5:** Final dental vulnerability scale

Dimension	Item	No (0)	Yes (1)
Overall Health	Does your health keep you from doing any of you daily activities?		
	Do you have any movement difficulties?		
	Do you have any disease that needs monitoring?		
Oral Health	Do you consider it important to take care of your mouth?		
	Do you believe that mouth diseases can be avoided?		
	Do you consider yourself responsible for your oral health?		
	Do you consider it important to have all the teeth in your mouth?		
Infrastructure	Do you have a bathroom in your house?		
	Do you have electric power in your house?		
	Do you have running water in your house?		
	Do you have sewage collection at your house?		
Healthcare Services	Are you familiar with the public primary care unit where you can visit?		
	Do you use a public primary care unit?		
	Are you accompanied by an oral health team?		
	Do you see the dentist without paying?		

## Discussion

The Dental Vulnerability Scale developed based on evidence of content and internal structure validity, showed four dimensions (overall heath, oral health, infrastructure, and healthcare services) and 15 distributed items.

Usually the development of a measuring instrument is based on theory/concept [62] and since the literature still has no instrument capable of exclusively measuring dental vulnerability [63], we adopted the concept defined as being a set of factors of the social, structural, general, mental and oral dimensions, in addition to health services and public management that influence the dynamics of the health-disease process in dentistry. The DVS proposed corroborates the concept described above, since the individual components are represented by the dimensions of overall health and oral health; the social or collective component by the dimension of the infrastructure; and lastly, the institutional component by the dimension of healthcare services.

The scales are developed when one seeks to measure a phenomenon that is not directly quantifiable, but a theory or concept points to its existence [20]. Certain indicators related to items of the scale allow obtaining a reasonably precise measurement of the phenomenon [33]. Therefore, the validation of a scale, which is a process in which we gather and evaluate the evidence to support the adequacy, significance, and usefulness of decisions and inferences, will validate the scale when it truly measures what it propose to measure, and for this, we seek the evidence of validity of the instrument items [33, 64].

Some instruments are widely used in Brazil, as the Oral Health Impact Profile (OHIP) [65], and the Coelho and Savassi Family Risk Scale was developed with the purpose of organizing home visits, based on the attribution of scores for sentinels considered relevant that were present in the SIAB. Despite presenting a sensitivity for defining families that require greater health care [16], this instrument has some limitations, such as difficulty in obtaining updated information, since studies have confirmed that updating at SIAB does not occur regularly [66], besides considering the risk for the family and not the individual, which can place a person within conditions not belonging to them. Other limitation is the scale does not have structure validity, and therefore, we cannot affirm if its items correlate, and if the scores are attributed in an adequate manner. Additionally, the sentinels encompass many families, classifying many of them as high risk, which hinder collection of concrete vulnerability data. Another issue is that there is no information about oral health in this scale; thus, we cannot use it to measure dental vulnerability [16, 66].

Studies addressing individual risk relative to oral health are scarce in the literature, making a comparative analysis difficult. This is the first study to prepare an instrument capable of measuring dental vulnerability, with characteristics that cover the entire national territory and with sufficient evidence of internal structure validity.

Evidence shows the instrument is multidimensional, comprising overall health, oral health, infrastructure, and healthcare services dimensions. As to the variables observed in the instrument after confirmatory factor analysis, we observed a greater relation between the “infrastructure” and “healthcare services” dimensions, and that the “overall health” dimension proved to be independent. This study showed that all proposed dimensions, present in the final fifteen items, may be appropriate to measure dental vulnerability in individuals from all Brazilian regions.

As to the items of overall health present in the DVS, it is necessary to ponder over the principle of integrality of individual care in the process of work, aiming at production of knowledge and new practices required in their care, for example, in chronic diseases, such as diabetes, hypertension, and obesity, which are very prevalent in the Brazilian population [67].

To consider systemic monitoring conditions as items of the scale is consistent with the literature. Obesity, for example, is a risk factor for innumerable systemic diseases, and there is a possible association between obesity and oral diseases [68, 69]. In the same way, diabetes, a morbidity that requires medical and dental follow-up, is related to dental vulnerability [70].

Additionally, hypertension, which is a chronic multifactorial disease that affects approximately 1.5 billion persons all over the world, appears or worsens in association with modification of oral conditions (gingivitis, periodontitis, and tooth loss) [71–73]. On the other hand, only clinical evaluations are not sufficient to measure health of the individual and identify vulnerable population groups, reinforcing the importance of using instruments that rate the perception of the individuals of their health and oral health [74].

The items related to oral health deal with the individual’s self-perception, which is determined by a need perceived as a result of their health condition and previous knowledge of the disease [75]. It can be considered a subjective indicator of the oral health condition that is strongly associated with the pattern of demand for dental services and the definition of priorities for planning, aiming to reduce inequities [76, 77]. When one observes the characteristics of participants of this study, the index that measures the prevalence of decays in adults and children (DMFT and DEFT, respectively) was at a mean of 11 for adults and 3.5 for children, which is considered very high. Despite a decline in prevalence of caries in Brazil, some studies have pointed out that the caries disease is disproportionate in reaching the economically vulnerable population. They also provide knowledge of how the epidemiology of this disease occurs in the different groups, which is fundamental for establishing preventive strategies of control [78–80].

Self-perception in oral health, validated as an instrument needed in treatment, is a negative self-evaluation predictor, and can also be related to a lower per capita family income and lower level of education [76, 77, 81]. Thus, self-perception can be used to identify individuals more or less vulnerable [77, 81] and who need treatment, thus corroborating its use in items of the DVS (“Do you consider it important to take care of your mouth?”; “Do you believe that oral diseases can be avoided?”; “Do you consider yourself responsible for your oral health?”; and “Do you consider it important to have all the teeth in your mouth?”.

Living conditions are strongly linked to quality of life and permeate the social inequalities present in the communities, gaining strength as one of the determining factors of the levels of health in a population [82]. Within the Brazilian territorial scenario, extensive inequality is noted as to housing, which points out the fact that the vast majority of the Brazilian population lives in houses that are not appropriate or insufficient for residential density [83], Adding this to the absence of housing conditions ideal for promoting health and well-being, as per the results found in this study, in which the variables related to living conditions within the infrastructure dimension, were consolidated as being important for defining dental vulnerability [4].

In this study, the infrastructure criteria of “presence/absence of a bathroom/electric power/running water and sewage system” remained in the DVS scale, reinforced by the literature, which indicates the lack of infrastructure services (without, at least, one of the basic services: electric power, general water mains with internal plumbing, general sewage system or septic tank, and garbage collection) compose the criteria of the household inadequacy indicators, which are determining and conditioning factors that interact in the life of the population, interfering in health promotion [84].

Among the items belonging to the infrastructure dimension, sanitation is considered by the World Health Organization as the main element of environmental health, with a significant role in the prevention and promotion of health of the population. Unfortunately, some items such as the absence of electric power, absence of sewage system and of running water are still a common reality in all Brazilian states, characterizing the precariousness of basic sanitation services [85].

The items of “healthcare services” dimension may indicate a high vulnerability of the population to oral diseases when there is no well-structured PHC service, with difficulty in scheduling visits. Access is related to the various possibilities of entering healthcare services, which would be involved with the location of the health facility, the availability and flexibility of schedules and the days the facility serves, the possibility of attending non-scheduled visits and the user's self-perception about the quality of access [86–88].

There is agreement on the advantages of PHC-based health systems: increasing the population's access to services is related to better performance in disease prevention and health promotion, improved health levels, reduced health inequities, and better awareness of chronic diseases among the population [89, 90].

Integrating OHT to FHS has a positive impact in increasing the access of families to curative and preventive procedures in health facilities [87]. The item "Are you accompanied by an oral health team?" is related to the access to dental services, and reflects the influence of individual and contextual determinants on the quality of oral health care. The characteristic of this access (periodicity, ease of scheduling) can be an important indicator of how the major oral health problems have been identified and followed up. It is worth noting that integrating OHT to FHS is heterogeneous throughout the national territory. Access to the dentist in certain regions results in decrease and relief of pain and suffering, not characterizing in a single way a prevention factor for oral diseases [91].

This aspect becomes even more relevant so that in the future it can assess classifications with scores, allowing professionals to use a safety and precision scale in the way of interpreting the instrument's scores.

With regard to free dental care ("Do you see the dentist without paying?"), individual income and the ability to pay for services are constantly pointed out as important factors in access to healthcare services [92]. An analysis of the use of dental services showed that in the previous 12 months, the richer individuals consulted dentists 2.8 times more than the poorest [93].

As a limitation of this study, it is important to consider the internal structure phase had its collection centered on a demographic region of the city of São Paulo, requiring expansion and testing in other scenarios. Thus, this limitation also imposes restrictions for the standardization and for the creation of the instrument's score table. So, at this point, the use of EVO should be restricted to research applications. Another aspect is the application of this scale by a large and varied number of individuals, which may make it difficult to standardize the application of the questionnaire, although barriers to avoid lack of standard have been implemented throughout this study.

## Conclusion

This study shows that 15 items structured in four dimensions are sufficient to measure dental vulnerability. The DVS presents evidence of satisfactory validity, indicating its feasibility to be used by health professionals, students, and managers to plan actions and services related to oral health at PHC.

Since content validity was carried out in the national context and the internal structure concentrated in a region of Greater São Paulo, in future research the application is necessary in other regions of Brazil, expanding the evidence of validity of the internal structure, as well as studies and evidence other variables (such as the user's health indicators), and other instruments.

## Data Availability

The datasets used and/or analysed during the current study are available from the corresponding author on reasonable request.

## Abbreviations

AERA: American Educational Research Association
AGFI: Adjusted goodness of fit index
APA: American psychological association
CFA: Confirmatory factor analysis
CFI: Comparative fit index
CVR: Content validity ratio
DEFT: Decayed, extracted, filled teeth (adult)
DMFT: Decayed, missing, filled teeth (children)
DVS: Dental vulnerability scale
EFA: Exploratory factor analysis
FHS: Family health strategy
GBL: Greatest lower bound
GFI: Goodness of fit index
GOFs: Goodness-of-fit
IBGE: Brazilian Institute of Geography and Statistics
ICF: Informed consent form
KMO: Kaiser-Meyer-Olkin
MH/BR: Ministry of Health—Brazil
MRFA: Minimun rank factor analysis
NCME: National Council on Measurement in Education
NNFI: Non-normed fit index
OHIP: Oral health impact profile
OHT: Oral health teams
PA: Parallel analysis
PCU: Primary care units
PHC: Primary health care
RMSEA: Root mean square error of approximation
RMSR: Root mean square of residuals
RPA: Robust parallel analysis
SBIBAE: Sociedade Beneficente Israelita Brasileira Albert Einstein
SIAB: Primary care information system
SMS/PMSP: Municipal Department of Health of São Paulo
ULS: Unweighted least squares

## References

[1] Manfredini MA, Narvai PC (2018). Concepções de lideranças de saúde sobre saúde bucal e controle de políticas públicas. Rev da ABENO.

[2] Corrêa GT, Celeste RK. Associação entre a cobertura de equipes de saúde bucal na saúde da família e o aumento na produção ambulatorial dos municípios brasileiros, 1999 e 2011. 2015;31(12):2588–98. 10.1590/0102-311X00000915 26872235

[3] Pucca Junior GA, Gabriel M, Almeida Carrer FC de, Paludetto Junior M, Lucena EHG de, Melo NS de. Acesso e cobertura populacional à saúde bucal após a implantação da Política Nacional de Saúde Bucal “Brasil Sorridente.” Tempus Actas de Saúde Coletiva [Internet]. 2020 Jul 3 [cited 2020 Aug 11];14(1):29–43. http://tempusactas.unb.br/index.php/tempus/article/view/2629

[4] Censo Demográfico | IBGE [Internet]. [cited 2020 Feb 5]. https://www.ibge.gov.br/estatisticas/sociais/populacao/9662-censo-demografico-2010.html?edicao=9673&t=sobre

[5] Carnut L, Filgueiras LV, Figueiredo N, de Goes PSA (2011). Validação inicial do índice de necessidade de atenção à saúde bucal para as equipes de saúde bucal na estratégia de saúde da família. Cienc e Saude Coletiva.

[6] Aguiar SFA, da Rocha MP (2019). Políticas de Saúde Bucal no Brasil: Mudanças a Partir de 1988. ID line. Rev Psicol.

[7] Dimenstein M, Cirilo NM (2020). Abordagens conceituais da vulnerabilidade no âmbito da saúde e assistência social. Pesqui e Práticas Psicossociais [Internet].

[8] Ayres JR de CM, Calazans GJ, Filho HCS, França-Júnior I. O risco, vulnerabilidade e práticas de prevenção e promoção da saúde. Saúde em debate. 2003;(170):375–417.

[9] Carmo MED, Guizardi FL (2018). O conceito de vulnerabilidade e seus sentidos para as políticas públicas de saúde e assistência social. Cad Saude Publica.

[10] Mendes JM (2018). Risco, vulnerabilidade social e resiliência: conceitos e desafios. Rev Gestão Sustentab Ambient.

[11] Loh LW (2017). The importance of recognizing social vulnerability in patients during clinical practice. J Heal Care Poor Underserved.

[12] Nguyen VH, Lin SC, Cappelli DP, Nair S. The association between dental, general, and mental health status among underserved and vulnerable populations served at health centers in the US. J Public Health Dent [Internet]. 2018;78(1):41–8. 10.1111/jphd.12234 10.1111/jphd.12234 28719064

[13] Neves RG, Flores TR, Duro SMS, Nunes BP, Tomasi E (2018). Tendência temporal da cobertura da Estratégia Saúde da Família no Brasil, regiões e Unidades da Federação, 2006–2016. Epidemiol e Serv saude Rev do Sist Unico Saude do Bras.

[14] Peres Neto J, Mendes KLC, Wada RS, Sousa MSDLRD (2017). Relação entre classificações de risco utilizadas para organização da demanda em saúde bucal em município de pequeno porte de São Paulo, Brasil. Cien Saude Colet.

[15] Schwendicke F, Giannobile WV. Research for Prevention of Oral/Dental Diseases: How Far Have We Come? J Dent Res [Internet]. 2020 Jan 20 [cited 2020 Feb 2];99(1):5–7. 10.1177/0022034519889054 10.1177/0022034519889054 PMC6927065 31859587

[16] Coelho FLG, Savassi LCM (2004). Aplicação de Escala de Risco Familiar como instrumento de priorização das Visitas Domiciliares. Rev Bras Med Família e Comunidade.

[17] Savassi LCM, Lage JL, Coelho FLG (2012). Sistematização de um instrumento de estratificação de risco familiar: Escala de risco familiar de Coelho-Savassi. J Manag Prim Heal Care..

[18] Menezes AHR, Cardelli AAM, Vieira GB, Martins JT, Fernandes MV, Marrero T-L (2012). Classificação do risco familiar segundo escala de Coelho e Savassi – um relato de experiência Ciência. Cuid e Saúde.

[19] American Educational Research Association., American Psychological Association., National Council on Measurement in Education., Joint Committee on Standards for Educational and Psychological Testing (U.S.). Standards for educational and psychological testing. 2014

[20] DeVellis RF (2017). Scale development: theory and applications.

[21] Bardin L. Análise de Conteúdo. 2010th ed. Lisboa (Portugal): vol. 70; 1977. 225 p.

[22] Goodman LA (1961). Snowball sampling. Ann Math Stat.

[23] Lawshe CH. A quantitative approach to content validity. Pers Psychol [Internet]. 1975 Dec [cited 2020 Jan 25];28(4):563–75. 10.1111/j.1744-6570.1975.tb01393.x

[24] Lynn MR (1986). Determination and quantification of content validity. Nurs Res.

[25] Epstein J, Santo RM, Guillemin F (2015). A review of guidelines for cross-cultural adaptation of questionnaires could not bring out a consensus. J Clin Epidemiol.

[26] Gilbert GE, Prion S (2016). Making sense of methods and measurement: Lawshe’s content validity index. Clin Simul Nurs.

[27] Fundação Seade. Sistema Seade de Projeções Populacionais | Fundação Seade [Internet]. [cited 2020 Aug 7]. http://produtos.seade.gov.br/produtos/projpop/

[28] Ministério da Saúde. Brasil. SIAB [Internet]. [cited 2020 Aug 7]. http://www2.datasus.gov.br/SIAB/index.php?area=01

[29] Critério Brasil - ABEP [Internet]. Associação Brasileira de Empresas de Pesquisa. [cited 2020 Jan 26]. http://www.abep.org/criterio-brasil

[30] Brasil. Ministério da Saúde. Ficha de Cadastro individual e-SUS Atenção Básica [Internet]. [cited 2020 Jan 26]. (versão 2.1). http://189.28.128.100/dab/docs/portaldab/documentos/Cadastro_Individual.pdf

[31] Briggs NE, MacCallum RC (2003). Recovery of weak common factors by maximum likelihood and ordinary least squares estimation. Multivariate Behav Res.

[32] Costello AB, Osborne JW. Best practices in exploratory factor analysis: four recommendations for getting the most from your analysis. Pract Assessment, Res Educ [Internet]. 2005;10:1–9. http://citeseerx.ist.psu.edu/viewdoc/download?doi=10.1.1.110.9154&rep=rep1&type=pdf

[33] Hair JF, Black WC, Babin BJ, Anderson RE. Univariate data analysis. exploratory data analysis in business and economics. 2013

[34] Howard MC (2016). A review of exploratory factor analysis decisions and overview of current practices: what we are doing and how can we improve?. Int J Hum Comput Interact.

[35] Timmerman ME, Lorenzo-Seva U (2011). Dimensionality assessment of ordered polytomous items with parallel analysis. Psychol Methods.

[36] Kaiser HF. The application of electronic computers to factor analysis. Educ Psychol Meas [Internet]. 1960 Apr 2 [cited 2020 Jan 25];20(1):141–51. 10.1177/001316446002000116

[37] Ferrando PJ, Lorenzo-Seva U. Unrestricted item factor analysis and some relations with item response theory [Internet]. Tarragona; 2013 [cited 2020 Jan 25]. http://psico.fcep.urv.cat/utilitats/factor/

[38] Hayton JC, Allen DG, Scarpello V. Factor Retention Decisions in Exploratory Factor Analysis: a Tutorial on Parallel Analysis. Organ Res Methods [Internet]. 2004;7(2):191–205. 10.1177/1094428104263675

[39] Green SB, Redell N, Thompson MS, Levy R. Accuracy of revised and traditional parallel analyses for assessing dimensionality with binary data. Educ Psychol Meas [Internet]. 2016 [cited 2020 Jan 25];76(1):5–21. 10.1177/0013164415581898 10.1177/0013164415581898 PMC5965575 29795854

[40] Hendrickson AE, White PO (1964). Promax: a quick method for rotation to oblique simple structure. Br J Stat Psychol.

[41] Tabachnick BG, Fidell LS, Ullman JB. Using multivariate statistics

[42] Sivo SA, Xitao FAN, Witta EL, Willse JT (2006). The search for “optimal” cutoff properties: fit index criteria in structural equation modeling. J Exp Educ.

[43] Asparouhov T, Muthén B. Simple second order chi-square correction. 2010.

[44] Cronbach LJ. Coefficient alpha and the internal structure of tests. In: Psychometrika. Springer; 1951, pp. 297–334.

[45] McDonald RP. Test theory : a unified treatment. L. Erlbaum Associates; 1999. 485 p.

[46] Jackson PH, Agunwamba CC (1977). Lower bounds for the reliability of the total score on a test composed of non-homogeneous items: I: algebraic lower bounds. Psychometrika.

[47] Cortina JM. What is coefficient alpha? An examination of theory and applications. J Appl Psychol [Internet]. 1993 [cited 2019 Jun 25];78(1):98–104. 10.1037/0021-9010.78.1.98

[48] Vaske JJ, Beaman J, Sponarski cc. rethinking internal consistency in cronbach’s alpha. Leis Sci [Internet]. 2017 [cited 2019 Jun 25];39(2):163–73. 10.1080/01490400.2015.1127189

[49] Trizano-Hermosilla I, Alvarado JM. Best alternatives to cronbach’s alpha reliability in realistic conditions: congeneric and asymmetrical measurements. Front Psychol [Internet]. 2016 [cited 2020 Jan 25];7. 10.3389/fpsyg.2016.00769/abstract 10.3389/fpsyg.2016.00769 PMC4880791 27303333

[50] Schmitt N (1996). Uses and abuses of coefficient alpha. Psychol Assess.

[51] Panayides P (2013). Coefficient alpha: interpret with caution. Eur J Psychol.

[52] IBM SPSS Inc. SPSS Statistics for Windows, Version 23.0. IBM Corp Released 2015. 2015.

[53] Bado FMR, Rebustini F, Jamieson L, Cortellazzi KL, Mialhe FL (2018). Evaluation of the psychometric properties of the Brazilian version of the Oral Health Literacy Assessment in Spanish and development of a shortened form of the instrument. PLoS ONE.

[54] Lorenzo-Seva U, Ferrando PJ (2013). FACTOR 9.2: a comprehensive program for fitting exploratory and semiconfirmatory factor analysis and IRT models. Appl Psychol Meas.

[55] Wilson FR, Pan W, Schumsky DA (2012). Recalculation of the critical values for Lawshe’s content validity ratio. Meas Eval Couns Dev.

[56] Ayre C, Scally AJ (2014). Critical values for Lawshe’s content validity ratio: revisiting the original methods of calculation. Meas Eval Couns Dev.

[57] Baghestani AR, Ahmadi F, Tanha A, Meshkat M (2019). Bayesian critical values for Lawshe’s content validity ratio. Meas Eval Couns Dev.

[58] Serra F (2019). Construtos na pesquisa em estratégia: definição e clareza. Iberoam J Strateg Manag.

[59] Fokkema M, Greiff S (2017). How performing PCA and CFA on the same data equals trouble. Eur J Psychol Assess.

[60] Ferrando PJ, Lorenzo-Seva U (2018). Assessing the quality and appropriateness of factor solutions and factor score estimates in exploratory item factor analysis. Educ Psychol Meas.

[61] Brown TA. Confirmatory factor analysis for applied research, Second Edition—Timothy A. Brown - Google Livros [Internet]. 2015. https://books.google.com.br/books?hl=pt-BR&lr=&id=JDb3BQAAQBAJ&oi=fnd&pg=PP1&ots=-0ItdY3B_c&sig=l9s73c9yTKBuiXzESA-6TOvNfCk&redir_esc=y#v=onepage&q&f=false

[62] Dimitrov DM, Tenko R (2003). Validation of cognitive structures: a structural equation modeling approach. Multivariate Behav Res.

[63] Nora CRD, Zoboli E, Vieira MM (2018). Validação por peritos: importância na tradução e adaptação de instrumentos. Rev Gaúcha Enferm.

[64] Messick S (1979). Teste validity and the ethics of assessment.

[65] Possebon AP da R. Análise fatorial exploratória e confirmatória do instrumento OHIP-Edent. Universidade Federal de Pelotas. Universidade Federal de Pelotas; 2017.

[66] Madureira VSF, Weber A, Albrecht CC, Fávero DC, de Oliveira R de CF. Potencialidade e fragilidade da aplicação da escala de Coelho-Savassi: o olhar de estudantes de enfermagem. Supl Rev Saúde em Redes ISSN 2446–4813 v2 n1, Supl 2016 [Internet]. 2015 [cited 2020 Feb 5]. http://conferencia2016.redeunida.org.br/ocs/index.php/congresso/2016/paper/view/2868

[67] Ferreira AP de S, Szwarcwald CL, Damacena GN. Prevalência e fatores associados da obesidade na população brasileira: estudo com dados aferidos da Pesquisa Nacional de Saúde, 2013. Rev Bras Epidemiol. 2019 [cited 2020 Feb 5];22. http://www.scielo.br/scielo.php?script=sci_arttext&pid=S1415-790X2019000100420&tlng=pt 10.1590/1980-549720190024 30942330

[68] Chaffee BW, Weston SJ (2010). Association between chronic periodontal disease and obesity: a systematic review and meta-analysis. J Periodontol.

[69] Nascimento GG, Seerig LM, Vargas-Ferreira F, Correa FOB, Leite FRM, Demarco FF (2013). Are obesity and overweight associated with gingivitis occurrence in Brazilian schoolchildren?. J Clin Periodontol.

[70] Silva AM, Vargas AMD, Ferreira EF, de Abreu MHNG (2010). A integralidade da atenção em diabéticos com doença periodontal. Cienc e Saude Coletiva.

[71] Oliveira EJP, Rocha VFB, Nogueira DA, Pereira AA (2018). Qualidade de vida e condições de saúde bucal de hipertensos e diabéticos em um município do Sudeste Brasileiro. Cien Saude Colet.

[72] Boff E, Palma LZ. Avaliação da pressão arterial no atendimento odontológico como forma de busca ativa de novos casos de hipertensão arterial sistêmica. 2018.

[73] Mussane RD, Benedito FC da S, Joaquim DC, da Silva N ghalna, Rodrigues JC, Leite AKR de M, Leite ACR de M. Pacientes hipertensos: dos cuidados em saúde ao conhecimento das patologias orais e sua relação com a hipertensão arterial sistêmica | Mussane | Revista Diálogos Acadêmicos [Internet]. Revista Diálogos Acadêmicos. 2017 [cited 2020 Feb 5]. http://revista.fametro.com.br/index.php/RDA/article/view/133

[74] Passos-Soares JDS, Santos LPDS, Gomes-Filho IS, Neves MR, Cruz SSD, Santos PNP, Silva ICOD, Balinha IDSCE, Trindade SC. Impacto da perda dentária na qualidade de vida relacionada a saúde bucal de adultos. Rev Ciências Méd Biol. 2018;17(2):158.

[75] Barata RB. Acesso e uso dos serviços de saúde: considerações sobre os resultados da Pesquisa de Condições de Vida 2006. Vol. 22. São Paulo; 2008.

[76] Nascimento AR do, Andrade FB de, César CC. Validade e utilidade da autopercepção de necessidade de tratamento odontológico por adultos e idosos. Cad Saude Publica [Internet]. 2015;31(8):1765–74. http://www.scielo.br/scielo.php?script=sci_arttext&pid=S0102-311X2015000801765&lng=pt&tlng=pt 10.1590/0102-311X00150214 26375654

[77] Dalazen CE, Bomfim RA, De-Carli AD (2018). Fatores associados à autopercepção da necessidade de tratamento odontológico e de prótese em idosos brasileiros. Cien Saude Colet.

[78] Queiroz F de S, Costa LED, Santos KLS, Simões TMS, Silva PV da. Cárie dentária e fatores associados em crianças de 5 anos de idade do município de Patos-PB. Arch Heal Investig. 2018;7(5). 10.21270/archi.v7i5.2993

[79] Ely HC, Abegg C, da Rosa AR, Pattussi MP (2014). Redução da cárie dentária em adolescentes: distribuição temporal e espacial em 36 municípios do Sul do Brasil 2003 e 2011. Epidemiol Serviços Saúde.

[80] Jensen T, Vieira M, Scutti CS (2017). Comparação entre o risco social e o risco de cárie em famílias em situação de vulnerabilidade. Rev da Fac Ciências Médicas Sorocaba..

[81] Moura C, Gusmão ES, Santillo PMH, Soares RDSC, Cimões R (2014). Autoavaliação da saúde bucal e fatores associados entre adultos em áreas de assentamento rural, Estado de Pernambuco, Brasil. Cad Saude Publica.

[82] Alves J, Cavenaghi S (2016). Déficit Habitacional, Famílias Conviventes E Condições De Moradia. Séries Demográficas.

[83] Moulin A, Santos SP, Gomes M, Medeiros P, Luft RM. Direito à moradia: um direito social em construção no Brasil -social no Rio de Janeiro [Internet]. 2016 [cited 2020 Feb 5]. Available from: http://repositorio.ipea.gov.br/handle/11058/6575

[84] Viana RDM, de Souza CCA, Franco MPV, Souza LDM, De Miranda-Ribeiro A (2019). Carências Habitacionais no Brasil e na América Latina: o papel do ônus excessivo com aluguel urbano. Cad Geogr.

[85] Neto AK, Dos Anjos GM, Brandolff RDS, Goés TP, Da Silva JF (2017). Fatores relacionados à saúde pública e ao saneamento básico em comunidade rural de Barreiras, Bahia, Brasil. Rev Baiana Saúde Pública.

[86] Assis MMA, de Jesus WLA (2012). Acesso aos serviços de saúde: abordagens, conceitos, politicas e modelo de análise. Cienc e Saude Coletiva.

[87] Palacio DDC, Vazquez FDL, Ramos DVR, Peres SV, Pereira AC, Guerra LM, Cortellazzi KL, Bulgareli JV (2014). Evolution of post-deployment indicators of oral health on the Family Health Strategy. Einstein (Sao Paulo).

[88] Ramos DVR, Miraglia JL, Monteiro CN, Borchardt D, Tribis L, Sanchez TP, Bonfim D, Palacio DDC, Rosário MDL, Mota MJBDB. Risk assessment for oral urgent treatment in Primary Healthcare : a cross-sectional study. BMC Heath Serv Res. 2020;2:1–7. 10.1186/s12913-020-05859-2 PMC7643441 33148246

[89] Tesser CD, Norman AH, Vidal TB (2018). Acesso ao cuidado na Atenção Primária à Saúde brasileira: situação, problemas e estratégias de superação. Saúde em Debate.

[90] Brandão JRM, Gianini RJ, Novaes HMD, Goldbaum M (2011). The family health system: analysis of a health survey in São Paulo, Brazil. J Epidemiol Community Health.

[91] Peres MA, Latorre MDRDO, Sheiham A, Peres KG, Barros FC, Hernandez PG, Maas AMN, Romano AR, Victora CG (2003). Determinantes sociais e biológicos da cárie dentária em crianças de 6 anos de idade: um estudo transversal aninhado numa coorte de nascidos vivos no Sul do Brasil. Rev Bras Epidemiol.

[92] Chiavegatto Filho ADP, Wang Y-P, Malik AM, Takaoka J, Viana MC, Andrade LH. Determinants of the use of health care services: multilevel analysis in the Metropolitan Region of Sao Paulo. Rev Saude Publica. 2015;49(0). http://www.scielo.br/scielo.php?script=sci_arttext&pid=S0034-89102015000100301&lng=en&tlng=en 10.1590/S0034-8910.2015049005246 PMC4386561 25741652

[93] Barros AJD, Bertoldi AD (2002). Desigualdades na utilização e no acesso a serviços odontológicos: uma avaliação em nível nacional. Cien Saude Colet.

