# Correction to: Dental vulnerability scale in primary health care: evidence of content and structure internal validity

**DOI:** 10.1186/s12903-021-01801-y

**Published:** 2021-09-09

**Authors:** Danielle da Costa Palacio, Flavio Rebustini, Daniele Boina de Oliveira, João Peres Neto, Wander Barbieri, Thais Paragis Sanchez, Ana Carolina Cintra Nunes Mafra, Daiana Bonfim, Camila Nascimento Monteiro, Valmir Vanderlei Gomes Filho, Danielle Viana Ribeiro, Leandro Marsico Loschiavo, João Luiz Miraglia, Antonio Carlos Pereira

**Affiliations:** 1 Hospital Israelita Albert Einstein, Av. Albert Einstein 627, São Paulo, SP Brazil; 2 Faculdade de Odontologia de Piracicaba, Universidade Estadual de Campinas, Av. Limeira, 901 - Areião, Piracicaba , SP Brazil; 3 Ciências e Humanidades – Rua Arlindo Béttio, Universidade de São Paulo - Escola de Artes, 1000 - Ermelino Matarazzo, São Paulo, SP Brazil

## Correction to: BMC Oral Health (2021) 21:421 https://doi.org/10.1186/s12903-021-01742-6

After publication of the original article [1], the authors identified an error in the authors’ names of Danielle da Costa Palacio and Camila Nascimento Monteiro.

The incorrect authors’ names are:

Danielle da Costa: given name.

da Palacio: family name.

Camila Monteiro: given name.

Nascimento: family name.

The correct authors’ names are:

Danielle: given name.

da Costa Palacio: family name.

Camila: given name.

Nascimento Monteiro: family name.

The author group has been updated above and the original article [1] has been corrected.
